# “To die is better for me”, social suffering among Syrian refugees at a noncommunicable disease clinic in Jordan: a qualitative study

**DOI:** 10.1186/s13031-020-00309-6

**Published:** 2020-09-01

**Authors:** Lucy Maconick, Éimhín Ansbro, Sara Ellithy, Kiran Jobanputra, Mohammad Tarawneh, Bayard Roberts

**Affiliations:** 1 Department of Public Health and Policy, London School of Hygiene and Tropical Medicine, LSHTM, 15-17 Tavistock Place, London, WC1H 9SH UK; 2 Médecins sans Frontières, Amman, Jordan; 3 Médecins sans Frontières, London, UK; 4 Primary Care Director, Ministry of Health of Jordan, Amman, Jordan

**Keywords:** mental health, psychosocial, Humanitarian, conflict, Refugee, Jordan, Syria, non-communicable disease, Social suffering

## Abstract

**Background:**

The conflict in Syria has required humanitarian agencies to implement primary-level services for non-communicable diseases (NCDs) in Jordan, given the high NCD burden amongst Syrian refugees; and to integrate mental health and psychosocial support into NCD services given their comorbidity and treatment interactions. However, no studies have explored the mental health needs of Syrian NCD patients. This paper aims to examine the interaction between physical and mental health of patients with NCDs at a Médecins Sans Frontières (MSF) clinic in Irbid, Jordan, in the context of social suffering.

**Methods:**

This qualitative study involved sixteen semi-structured interviews with Syrian refugee and Jordanian patients and two focus groups with Syrian refugees attending MSF’s NCD services in Irbid, and eighteen semi-structured interviews with MSF clinical, managerial and administrative staff. These were conducted by research staff in August 2017 in Irbid, Amman and via Skype. Thematic analysis was used.

**Results:**

Respondents describe immense suffering and clearly perceived the interconnectedness of their physical wellbeing, mental health and social circumstances, in keeping with Kleinman’s theory of social suffering. There was a ‘disconnect’ between staff and patients’ perceptions of the potential role of the NCD and mental health service in alleviating this suffering. Possible explanations identified included respondent’s low expectations of the ability of the service to impact on the root causes of their suffering, normalisation of distress, the prevailing biomedical view of mental ill-health among national clinicians and patients, and humanitarian actors’ own cultural standpoints.

**Conclusion:**

Syrian and Jordanian NCD patients recognise the psychological dimensions of their illness but may not utilize clinic-based humanitarian mental health and psychosocial support services. Humanitarian agencies must engage with NCD patients to elicit their needs and design culturally relevant services.

## Introduction

Since it began in 2011, the conflict in Syria has resulted in the displacement of approximately 6.1 million people within Syria and over 6.5 million refugees into neighbouring countries, including Jordan [1]. The high burden of chronic non-communicable diseases (NCDs) such as diabetes, hypertension and cardiovascular disease among the affected population has been a key feature of the health sector response to the Syria crisis [2]. Humanitarian actors have limited experience in managing NCDs and have needed to develop specific tools, and programmatic and clinical guidance [3, 4].

Armed conflict has a profound impact on refugees’ mental health due to their exposure to violent and traumatic events, forced displacement and ongoing daily stressors [5–7]. There are surprisingly few reliable studies on the burden of mental disorders and psychological distress among Syrian refugees in Jordan, but evidence does suggest elevated levels of mental disorders among Syrian refugees in Turkey and Lebanon [8–10], while reduced functioning has been recorded among Syrian urban refugees compared to host populations in Jordan [11]. Guidance for mental health and psychosocial support (MHPSS) interventions to protect or promote psychosocial well-being and/or prevent or treat mental disorders in humanitarian emergency settings was developed in 2007 [12]. Specific MHPSS guidance has also been produced for the humanitarian sector in relation to the Syria crisis [12, 13].

People living with NCDs are at higher risk of mental health disorders in any context, due to direct effects on the brain or as a result of the disability, impaired functioning and chronic ill health related to the NCD [14]. Equally, mental disorders, such as depression, are also independent risk factors for development of poorer outcomes related to physical NCDs [14–16] and having a co-morbid mental disorder is associated with reduced help-seeking, poorer treatment adherence, and poorer prognosis for chronic physical conditions [17–21]. For conflict-affected populations, psychological trauma and the daily hardships of forced migration may increase their vulnerability to both mental and physical ill-health [22]. Physical NCDs may be impacted upon by the loss of control over daily life, financial difficulties, the breakdown of social networks and attendant harmful coping mechanisms, such as smoking and excess alcohol use [23–25].

*Social suffering* is a valuable explanatory framework to help understand mental and physical health needs [26]. It captures the close links between personal and societal problems and between individual and collective experiences to allow for a holistic view of health within a socio-political context [26, 27]. Analysis of individual narratives through the perspective of social suffering may shed light on how respondents make meaning of their health and broader conditions.

The advantages of integrating MHPSS and physical health services have gained increasing attention and MHPSS integration into general primary and secondary services is underway [28–31]. However, it is not clear how well the need to manage interconnected physical NCDs and mental health issues has been recognised, either in terms of the Syrian refugee response or more widely in humanitarian settings, and the evidence to guide such integrated responses is lacking. Given the psychosocial stressors and the rising global burden of NCDs impacting conflict-affected populations, this is an important gap. Designing relevant and effective interventions requires an understanding of both service users’ and providers’ perspectives. This paper aims to examine the interaction between physical and mental health of patients with NCDs at a Médecins Sans Frontières (MSF) clinic in Irbid, Jordan, in the context of social suffering.

## Methods

### Study setting

This paper is part of a wider evaluation, using the RE-AIM implementation framework, of the NCD services provided by the humanitarian agency MSF in Irbid, Jordan [32]. Almost 680,000 registered Syrian refugees fled to Jordan, of whom, 21% live in Irbid governorate, outside of formal refugee camp settings. MSF initiated the NCD service in 2014 in response to the high burden of NCDs and the barriers Syrians faced in accessing government health facilities. The service targeted patients with one or more of the following: cardiovascular disease, chronic respiratory disease or diabetes types 1 or 2 and provided free services, which included consultations by appointment, medications, laboratory testing and health education using context-adapted evidence-based clinical guidelines. While the majority were Syrian refugees, 30% of patients were from the Jordanian host population, in accordance with government requirements. A MHPSS service was incorporated in 2016 as staff recognised that patients’ MHPSS needs were significantly affecting their ability to engage with NCD care. The MSF service consisted of individual counselling and group psycho-education sessions delivered by trained national staff (Jordanian) counsellors with psychology qualifications, supported by an international supervisor, and it initially focused on mental health in relation to medication adherence. However, a significant burden of untreated mental health problems, ranging from psychological distress, anxiety, depression, psychosis, suicidality, and self-harm was identified by MSF staff that was not being addressed by their own or other available services. Therefore, the MSF service evolved to encompass a wider range of mental health needs.

### Study design and data collection

A qualitative study design was used, based on semi-structured interviews and focus group discussions conducted in August 2017. Adult patients attending NCD medical appointments were purposively selected to reflect different NCD diagnoses, genders, nationalities and those accessing different aspects of the NCD service. A convenience sample of patients from the waiting room was also invited to participate. Both Syrian and Jordanian patients were included, reflecting the fact that it is UNHCR policy to provide equitable access to MHPSS services to both host and forcibly displaced populations [33]. From a research perspective, it is important to understand potential differences in these populations’ attitudes or help seeking behaviour and how this may impact on care delivery. All patients interviewed attended the general MSF NCD service, while only one participant accessed the MHPSS component. A purposive sample of clinical, managerial and administrative staff was also interviewed, aiming to include staff from each cadre, national and international, past and present. National staff were all Jordanian, while supervisors and management staff tended to be international.

Topic guides for the semi-structured interviews and focus groups are presented in Supplementary Material 1. While these contained limited prompts on mental health, the interviews covered mental health in significant detail because participants focussed on it and interviewers then explored the issues raised.

Sixteen semi-structured interviews with adult NCD patients (eight women and eight men; six Jordanians and ten Syrians; all aged 60 or older, which was representative of the broader cohort), and two same-sex focus groups were conducted with a different group of eight female and eight male adult Syrian NCD patients (moderated by same-sex research assistants). All had NCDs (mainly hypertension and/or diabetes) and refugees had been in Jordan for between three and 5 years. Eighteen interviews were conducted with MSF staff from a range of clinical, managerial and support roles. See Supplementary Material 2 for further participant details.

The semi-structured interviews were conducted in Arabic by SE or a male research assistant or in English by EA, depending on participant language preference. Interviews were conducted at the MSF clinics (for patients) and at MSF offices or via Skype (for staff); one patient of the home visit service was interviewed at home. The interviews were audio recorded and field notes were taken. Arabic language interview transcripts were translated by research team members. Each translation was cross-checked by a second translator and by one of the Arabic-speaking interviewers.

### Analysis

A thematic analysis approach was applied using NVivo 11. Initial codes and themes were generated and revised in an iterative process. Patient interviews were analysed for differences in responses between genders and by nationality (Syrian vs. Jordanian). Staff interviews were analysed for differences in national versus international staff and MHPSS versus other clinical staff’s responses. Patient quotes were labelled according to the patient gender and country of origin for context. Staff quotes were not labelled in order to protect the confidentiality of staff respondents.

### Ethics

Written consent was obtained and information sheets were provided. A referral mechanism was in place for patients who expressed health or protection needs during interviews. Confidentiality was assured by use of private spaces for interview and removal of all identifying information from written documentation. Focus group participants were asked to respect the confidentiality of all participants, while being informed that the research team could not guarantee against residual disclosure. Ethical approval was provided by the Ministry of Health in Jordan, the MSF Ethics Review Board, and the Ethics Committee of the London School of Hygiene and Tropical Medicine.

## Results

Both Jordanian and Syrian patients described the interconnectedness of their mental and physical health, and demonstrated similar attitudes to mental health services. The Syrian patient accounts differed from Jordanian accounts with respect to describing the high burden of distress within their community, barriers to help seeking associated with displacement and their expectation that they should suffer from poor mental health as a result of their circumstances. Themes that emerged were similar for men and women. The findings of the qualitative analysis are described separately for patient and staff interviews, categorized into a coding tree (Supplementary Material 3).

### Patient interviews

#### Impact of social stressors and war experience on mental health

Daily stressors and their impact on the mental health of the individual and family were discussed by Jordanian and Syrian respondents of both sexes. These included economic hardship, lack of available employment and working restrictions (the latter were specific to Syrians). Male Syrian focus group participants explained:

‘*We do not have money, our finances are bad, and they all (other facilities) take money, only here (at the MSF clinic) they do not take money*.

‘*For us as refugees, you do not have any work chances, you only have to be young and work in construction; … work depends on physical powers, and we all (older people) here do not have this*.’

A Syrian woman described her financial worries and ways of coping:

*‘Sometimes poor finance makes me anxious, sometimes I keep all the night awake talking to myself from where I will get money … , so I … ask (our neighbor) to give me 5 (Jordanian dinar) and I tell him once I have money I will pay you back’*

Among Jordanians, those with low income described similar financial worries and, even those from a higher socioeconomic bracket reported that the affordability and accessibility of MSF care alleviated their financial concerns.

Both Syrians and Jordanians appeared to link social stress to their individual and family circumstances, while Syrian respondents also reflected on the suffering that the war in Syria had caused themselves and their wider community. Men and women expressed suffering in similar terms, such as anger, sadness, grief, hopelessness or even expressing a passive death wish. Both sexes described similar physical symptoms, which may be associated with psychological distress (tiredness, poor appetite and not feeling ‘comfortable’). The specific sources of suffering included concern for family members remaining in Syria, witnessed suffering or violence, displacement from home and, most prominently, separation and loss of family:

‘*When I am upset, I have trembles in my body, you know every one of us in different places, some people here, others still in Syria*,’ female Syrian patient.

*‘The mental status and the mood was better than here in Jordan … you know when one is in his country, things are different’*, male Syrian patient.

The collective suffering of the Syrian people was described by several Syrian respondents:

‘...*all the incidents that occurred in front of us, like killing and destroying. What should I say, something can’t be imagined by the brain … the suffering, which our people went through wasn’t witnessed by any other people in the earth*.’ male Syrian patient.

They perceived that others in their community experienced similar psychological, (‘*all people have anxiety, not only me*’) or physical manifestations of suffering (‘*we are all tired as Syrians. We were destroyed*’).

Further examples of the language used to describe mental distress are given in Supplementary Material 4.

#### Interconnectedness of physical and mental health

Both Syrian and Jordanian patients consistently described their health in terms of interconnected physical, psychological and social dimensions. Psychological distress, or specific traumatic events could trigger their illness, while “anger”, low mood or sadness could negatively impact on diabetes and hypertension control:.

*“My brother … had a heart attack and died, it was a shock for us … and since then I had hypertension. Then another brother died in 2005 and my husband died the same year … so these are the reasons behind having diabetes and hypertension,’* female Jordanian patient.

‘*I say my diabetes is not because of food … bad emotional status can increase the sugar level especially if the one is always tired*,’ male Syrian patient.

For Syrians, the psychological distress induced by their war and refugee experience was directly linked with their NCD condition:

‘*When I get sad and remember my sons in Syria … I keep crying and crying. Then my hypertension goes high or goes down … then I take a hypertension pill to settle down whenever I read some news about them*,’ female Syrian patient.

By contrast, patients of both nationalities perceived that “good psychological status” was necessary to avoid symptoms and that improving psychological health could benefit their physical health, possibly to a greater degree than medications.

‘*Let me tell you, I have tried when I am mentally comfortable, everything is good and it helps more than medication*.’ male Syrian patient

Patients of both nationalities described how social circumstances, mental health and physical health were closely interlinked. A Jordanian male respondent described this biopsychosocial relationship linearly, in that poor finances caused depression and sadness, which in turn worsened diabetes and hypertension. Echoing several other accounts, a male Syrian patient described how his own wellbeing impacted on the wider social network:

‘*When someone is surrounded by comfortable things, he has money and things good then he feels good and his life is happy, so he speaks with others and feels fine with them and communicates with them. … When anyone has love and trust with others, he’ll help him to stay in a good mood... if the family had bad mood … it affects the human’s health*,’ male Syrian patient.

#### Psychological distress impacting on engagement with NCD care

For Syrian patients, their social suffering translated into difficulties engaging with the healthy living advice provided at the NCD clinic. They described being too preoccupied with thoughts of Syria or feeling psychologically or economically ill-equipped to follow lifestyle advice:

*Interviewer: ‘has anyone of you changed his lifestyle after he found about his disease?’*

*Male focus group participant: ‘nothing changed … we don’t feel comfortable, always thinking about what happened at our country’*

*‘Because of circumstances I cannot follow this plan … I mean the psychological circumstances, I’m out of hard circumstances so all of this affects, and when the financial situation is difficult as well.’ male Syrian respondent.*

One female Syrian focus group participant expressed thoughts of death and lack of motivation to engage in self-care:

‘*Every time I come here, they advise me take care of yourself and I always tell them to die is better for me.’*

By contrast, Jordanian patients seemed well engaged with the NCD clinic and, in addition to its affordability and convenience, many attributed their good disease control to feeling “comfortable” due to the “care”, individual attention and respect they received from MSF staff. Staff explained the differences in engagement between Syrians and Jordanians in relation to psychological distress and lack of agency, as discussed further below.

#### Seeking help and health beliefs around psychological distress

Despite the majority of patients speaking openly about psychological distress and clearly linking it to the onset or exacerbation of their physical disease, they did not seek help for this distress at the MSF clinic. The main reason appeared to be a lack of awareness of the MSF MHPSS services among patients of both sexes and nationalities. Only two interviewees were aware of the services, with one attending individual counselling sessions and a second attending a waiting room psychoeducation session. Moreover, when MHPSS services were described to them, most appeared reluctant to engage. We identified a number of health beliefs that my have influenced this.

One key belief was that the MSF clinic was not the appropriate place to seek help for psychological difficulties. Patients appeared to believe that there was a separation between biomedical services and sources of help for psychological distress. They perceived the doctors’ role as focussed on interpreting clinical data and prescribing, as illustrated here:

‘ … if I told them (that I feel upset or can’t sleep), they don’t react because it’s not their business...their mission is to give me medication only,’ female Syrian patient.

Having control over biomedical parameters appeared to satisfy and relieve the patients. For example, despite a female Syrian patient becoming tearful when describing her family’s separation, (‘you can say that most of the days I feel upset, I’m so sensitive … when I am upset I have trembles in my body, you know every one of us in different place, some people here others still in Syria {patient began crying}’), she maintained that she did not need the MSF MHPSS services, since her biomedical care was in order: ‘my lab test results always good … so everything is OK’.

The second key belief was that mental health issues were a private matter. Patients of both nationalities agreed that they held no place in the biomedical model of care provided at the clinic, as illustrated by one Jordanian male, who expressed anxiety: ‘I do not think this (clinic) is a proper place (to seek help for psychological issues) … these are personal things.’

There was a sense also that Syrian respondents, in particular, sought to normalise their distress as similar to that of others in their community and therefore not a problem or illness amenable to a healthcare solution:

‘Honestly (I have complained about anxiety) for four years … This is the first time I tell... but what to tell about this, all people have anxiety not only me, everybody that comes here say he or she has anxiety, all people have anxiety and I am like them, what shall I do?’ female Syrian patient.

Since there was a perceived inevitability to their distress, which had known and inalterable root causes, males and female respondents of both nationalities questioned the utility and effectiveness of bringing these issues into the medical consultation:

*‘What would I tell them about this, and how could they help? … I have members of my family still in Syria, how do you think my psychological status will be, would I be comfortable...? So what would I say to them here, that I have family still in Syria, what would they do? Or how can they help? I only say thanks Allah for everything’* female Syrian patient.

*‘(I do not you tell them in the clinic if I feel upset since) the reasons for sleeplessness are known*.’ male Syrian patient.

Among the minority of respondents who were aware of MSF MHPSS services did not perceive it as useful, rather a passive counselling service where, as a male Syrian patient explained, “you explain to him your situation and he listens to you, so you express your feelings.’ One respondent implied that psychological symptoms were not “clear” and therefore not amenable to intervention by doctors, whose role, again, was to prescribe:

Interviewer: ‘*when you feel anxious or having sleeping disorders, do you share this with the staff at the clinic and ask for their help*?’

Patient: ‘*If the disease was clear we tell them and they give us medications, and if not … they tell us that they do not have medication for this disease*.’

Among the minority of patients who described alternative health seeking strategies to manage their distress, respondents of both nationalities reported relying on family or religion.

‘I *did not try (to talk to someone at the clinic about anxiety) because I have sons and daughters and I have good relation with them.*’ female Jordanian Patient

Several patients discussing distressing topics such as bereavement and loss concluded by referring to how Allah is ultimately the only source of a solution to their troubles:

‘*Our situation is miserable and we can’t have emotional comfort. Never. It’s our destiny to be in a big crisis and our problems can’t be solved except by Allah, (not) even the researcher, (or) even the doctor. All the people have problems. May Allah help all people … May Allah protect us all*,‘ female Syrian patient.

### Staff interviews

The findings from staff interviews we have included here focus on staff perceptions of the mental health of their patients, the barriers to patients accessing the MHPSS service, and the impact of mental ill-health on their engagement with physical NCD care. While the topic guide referred only briefly to these issues, all staff emphasised the high burden and impact of mental health problems amongst their Syrian patients and many contrasted their experiences with those of Jordanian patients. Multiple staff reported recent cases of patients requiring psychiatric intervention, some with suicidal ideation and others experiencing gender-based violence, which complicated NCD care delivery.

#### Interaction between mental health and physical health

The majority of staff respondents, including clinicians and counsellors, echoed patient beliefs that traumatic experiences and ongoing daily stressors were interlinked with patients’ physical health. These stressful experiences also impacted on patients’ engagement with care for physical NCDs. Staff emphasised Syrian patients’ exposure to violence, their loss of family and home, social isolation and the breakdown of traditional community structures. MHPSS staff in particular, reiterated patients’ belief in the causal link between psychological stress and the onset or exacerbation of their NCD condition:

*‘You find out that these patients not only suffer from hypertension, diabetes, they are refugees who lost almost everything, especially when they keep saying that they had these diseases because of their circumstances like (losing a child), and they blame themselves for everything’*.

One national staff member agreed with the patient perspective that, given the circumstances, suffering was inevitable among Syrians (‘ *… they were exposed to war and their current situation is very bad … they are refugees so they will have some sort of mental problems*.’).

Staff recounted the chronic daily stressors to which Syrian patients were exposed, including poverty and indebtedness; vulnerability to exploitation; crowded, poor-quality living conditions; and social isolation. Some considered financial pressures and the inability to work in Jordan as the key psychosocial challenge facing Syrians and that this impacted directly on their mental and physical health.

‘*The financial challenges, the social challenges, are the most important factor in anxiety existence and other psychological disorders for NCD patients, which can affect their medical readings for blood pressure and sugar …* ’

#### Psychological distress impacting on NCD care

They In contrast to patient accounts of the interlinked physical, psychological and social dimensions of health, one staff member felt that both patients (of either nationality) and MSF doctors were slow to acknowledge the psychological component of illness and the need to address it to successfully manage the physical component.

*‘Unfortunately I see that people are not capable of admitting that they have psychological aspect that would affect their bodies, you have to admit this and ask for help, inform the staff that I need someone to support me through psychological counselling or support.’*

They reported how financial hardship was both a source of distress and directly prevented patients of both nationalities engaging in lifestyle change such as formal exercise activities and healthy eating. Both national and international staff respondents emphasised that medication adherence was also negatively impacted by psychosocial issues related to Syrian patients’ war and refugee experience:

*‘To be honest the social side, the loss, the situations they have been in, affect them a lot. This is one of many things that affects their adherence to medications or treatment in general.’*

*‘And then … intimate partner violence … yeah, the lady has diabetes but … the reason she is not taking her medicine is … all these other home psychosocial factors …* ’

In addition to reducing adherence, non-medical national staff members perceived that psychological comorbidity directly impeded the effectiveness of NCD medications:

*“ … anxiety and other psychological disorders for NCDs patients, which can affect their medical readings … despite taking medications … the tension medications will not do their effect*.’

Several staff commented on the futility of promoting lifestyle change when their patients were dealing with traumatic war experiences:

*‘As I was hearing the stories I thought … this man’s problem is not that he’s smoking too much. His problem is that he … experienced sexual violence, physical violence in prison in Syria … these two are linked.*’

Jordanian national staff recounted how social stress was tied to Syrians’ psychological distress and illustrated the differences they experienced in providing care for Syrian refugees compared to Jordanians:

*‘ There isn’t really much traditional or cultural differences between me and Syrian patients because we come from the same area but mostly the social economic status is different, the lifestyle is different … let’s say the impact of their life situation it affects their disease and it affects the way they want to deal with these diseases.’*

Staff contrasted Syrians’ and Jordanians’ engagement with care and motivation to self-care, which many linked to the concept of ‘hope’. They reported that Syrian patients’ hopelessness for their future either in Syria or Jordan, their disempowerment and disengagement from their current existence in Jordan, and their lack of meaningful daily activity, all impacted on their motivation..

‘*This certain population of people (Syrians) they don’t have much hope in their future life so they don’t really...some of them they just don’t care about improving their status to be better because they think that life has ended since they left Syria*.’

In one striking example of this focus on the importance of ‘hope’, one interviewee described a young man’s lack of social support and harmful coping strategies, and how this created a cycle of deteriorating physical and mental health:

‘*If he has been used to a small village in rural environments, without support from family or society, he is inclined just to get very fed up, to get feelings of hopelessness, sitting in the apartment all day, going next door to smoke the shisha, by taking Shawarma in the corner shop, and just gets larger … and (these) aggravate the disease and hypertension, which in turn aggravate the feelings of hopelessness …* ’

By contrast, hope and engagement in the future, more common among Jordanians, was seen by one clinician as improving self-motivation and, therefore, clinical outcomes.

‘*I see some Jordanian patients their blood sugar readings are lower, their blood pressure is more controlled and most important they want to control their disease, they care to control their diseases and that is for me the main difference. They have something to look to … . they look forward to tomorrows, but the Syrians here, they don’t as much*.’

Once Syrians started to settle for the longer-term in Jordan, their outcomes improved, according to another staff member.

‘In the last six months maybe … maybe some patients find job or find house. And they leave the talk about the return to Syria … and they try to adapt for their new situation and to find a new solution for their life. So they want to change it now. The effect on their health.’

#### Seeking help from the MHPSS service and mental health beliefs

Staff’s perception that patients chose not to seek help from MSF’s MHPSS service was consistent with patient accounts. They perceived patients MHPSS care seeking behaviour as being influenced by awareness, sociocultural and economic factors as well as doctor-held and patient-held beliefs.

All staff, especially MHPSS staff, perceived that lack of awareness was the principal barrier to patients of both nationalities accessing MHPSS services. This was reportedly linked to a MSF national staff doctors’ lack of engagement with the service. Referrals to MHPSS services were initially made only via theses doctors. In explaining the initially very low referral rates, doctors cited their distrust in the quality and effectiveness of the service. It appeared that they believed that counsellors were encroaching on a doctors’ territory and that they should stick to a defined and limited role:

‘It is good to have the mental health department and the counsellors but sometimes they may diagnose and they may diagnose incorrectly and I ask them many times please don’t diagnose because you are not a psychiatrist, you are a counsellor and we are referring this patient to you for maybe CBT or for more psycho-social support more than diagnosing and suggesting medications.’

Doctors’ beliefs about the MHPSS service appeared to be linked to the prevailing medical culture. Service supervisors, who were all international staff, underlined the fact that mental health was traditionally the preserve of hospitals and specialists in Jordan. They perceived that national doctors compartmentalised physical and mental ill health and that this may explain why a large burden of psychological morbidity among the Irbid cohort was going unrecognised.

Sociocultural factors were also at play in the low referral rates. While not overtly mentioned in patient accounts, several staff reported that there was stigma associated with mental ill health among the patients and broader society, without distinguishing by nationality. National staff doctors described their own reluctance to label patients with mental health diagnoses and perceived a referral to the MHPSS service as unacceptable to patients since it labelled them ‘crazy’. Staff thus reported modifying their language around MHPSS referral (‘Patients think … it’s a psychiatric or something like that. We tell him it is for support’.) It appeared important to reassure patients they were not labelled as being different compared to others and that it was 'socially ok to go' to counselling services. It was notable from staff accounts that psychosocial interventions framed as “living well” with diabetes programmes, delivered by both health education and MHPSS staff who taught pragmatic skills, such as problem solving and inter-family communication, were well accepted by patients. A further influence on low referral rates may have been doctors’ belief that Syrian’s distress was a natural response to their circumstances, echoing patient beliefs.

Other barriers to patients’ MHPSS health seeking, irrespective of nationality, were also described. These included women’s limited autonomy, and need for a male family member to attend. This was noted as limiting their ability to engage around gender based violence issues. Socioeconomic factors, whereby patients would choose to spend limited household finances attending medical consultations and laboratory visits rather than attending MHPSS session were described by several staff. They perceived that patients placed less value on the MPHSS service compared to other clinical aspects of the MSF NCD service.

Staff recounted efforts made by the MSF team to address access barriers. They described running training sessions for all clinical staff and promoting dialogue between MHPSS staff and their medical colleagues. In addition, they expanded referral rights to nurses and social workers and engaged in awareness raising and promotion of self-referral through waiting room psycho-education sessions.

## Discussion

This study describes how Syrian refugees and members of the host community living with NCDs in Jordan connected their psychological and social suffering with their physical illnesses, yet most did not perceive the MSF NCD service as a space to address their mental health. We will discuss patient and provider accounts from the explanatory perspectives of the Syrian and Jordanian cultural context, social suffering and social hope. The latter are not new concepts but we apply them specifically to the experience of Syrian refugees in Jordan and in the context of a programme for physical NCDs delivered by a humanitarian actor [27, 34, 35]. We will then discuss operational implications for the humanitarian sector, given that little is known about how humanitarian agencies should best integrate mental health care into chronic disease services in culturally relevant ways.

### Interconnection of mental health, physical health and social circumstances as social suffering

There is plentiful evidence from high-income settings that the incidence of mental ill-health is higher in people with physical NCDs and vice versa. Furthermore, people with comorbid physical and mental illness experience poorer health outcomes, such as decreased medication adherence, greater functional impairment, and higher risks of complications and early mortality [14, 36, 37]. There is also an increasing body of evidence linking stressful life events or chronic perceived stress with the onset of physical NCDs, such as diabetes [38, 39]. However, what is clear from our study is how respondents also linked their physical and mental health with their social world and with social suffering specifically.

The concept of social suffering links both physical ill health with social problems and individual experience with collective experiences [26, 27]. In conflict settings, this implies that social, cultural, political, and economic issues are intertwined with matters of public health [40]. Kleinman has proposed social suffering as a “social theory of global health” with several important implications. These include the notions that socio-political and socio-economic factors are directly implicated in disease; that social or bureaucratic infrastructure, designed to manage disease, can actually cause or worsen suffering; that pain and suffering are not limited to the individual but may extend to the wider family or community network; and, finally, that conditions should be defined and addressed holistically, incorporating both health and social policy responses [26]. Consistent with this theory, participants in our study could not separate the social and political context, which was responsible for them suffering trauma, poverty and powerlessness, from their physical health [27, 41].

Patients of both nationalities and staff described psychological distress as a cause or exacerbating factor in physical ill health and vice versa. Whilst Jordanians made this link in relation to their individual circumstances, in these interviews Syrian respondents additionally linked their physical and mental health to the collective experience of their community. The Syrian experience of physical health in this setting occurred on a backdrop of structural violence linked to their displacement and refugee status in Jordan. The poverty and social disadvantage experienced by many Syrians in Jordan may have impacted *directly* on their physical NCDs, and this is echoed in findings from other contexts. For example, depression and diabetes have been found to intersect more frequently in low income populations because of the strong relationship between depression and poverty [42]. This may have been less prominent for Jordanian patients, whom staff reported were generally of higher socio-economic status and education level than Syrian patients.

The concept of social hope links resilience and wellbeing to social context and to access to resources, both at the individual and community level [27]. A contrast in social hope between Jordanian and Syrian patients was noted by staff and this may be linked both to Jordanian’s relative wealth and their rootedness. Those Syrians who had settled in Jordan and were able to access resources, such as work and housing, were seen as demonstrating greater social hope. This in turn was perceived as positively impacting patients’ NCD outcomes, through increasing motivation to change and to self-care. In contrast, many refugees were observed by staff as being in a state of entrapment, both as a result of the structural barriers to improving their health and their internal conflict about whether to accept living in Jordan or to continue to hope for eventual return to Syria despite their knowledge that their lives there had been destroyed. This ‘sense of entrapment, of having nowhere to go’ has been described by Hage as the enemy of social hope [43].

Hassan et al. explored the cultural context around mental health and psychosocial wellbeing of Syrians affected by the conflict [13]. The explanatory model offered by patients in our study is in keeping with the ‘sociocentric’ and ‘cosmocentric’ understandings of the person described by Hassan as common in the Syrian population [13]. From this perspective each individual is linked to every other creature created by Allah and there are two dimensions to every individual: the universal dimension governed by the will of God and the social dimensions governed by rules of conduct [13]. The patient accounts illustrate these concepts as applied to health, where health of the individual is affected by other people and events and, in turn, an individual’s health affects the whole family and community, which chimes with the theory of social suffering.

Our findings suggested that beliefs about mental health were similar between the Jordanian and Syrian participants. Similarities in cultural understandings of mental health are perhaps expected given that the Syrian and Jordanian populations living either side of the border near Irbid share close historical, trade and family ties [44].

### Health care seeking and uptake of MSF mental health and psychosocial services

A further key finding was that few interviewed patients of either nationality were aware the MHPSS service existed. Our larger service evaluation found that uptake of the MHPSS service was well below the perceived need and that patients referred to MSF MHPSS services often failed to attend [45]. They appeared to value MHPSS services less than physical health consultations and financially precarious families were willing to spend time and money to attend NCD medical consultations but not MHPSS appointments [45].

Health or care seeking behaviour has been defined as any action undertaken by individuals who perceive themselves to have a health problem or to be ill for the purpose of finding an appropriate remedy. Our findings imply that neither patients nor doctors perceived that the psychological distress patients experience was a ‘health problem’. Rather, it was “normal” for Syrians, in particular, to experience psychological distress given their circumstances and to attend the MHPSS service branded them as unacceptably “abnormal”. Second, the biomedical setting of the clinic was an inappropriate venue to seek help for psychoscocial issues, perhaps because of a misconception as to what the service offered. Many existing psychiatric services in Jordan favour a medication based approach, which is in contrast to the talking therapies offered by the MSF MHPSS [46]. Third, the MHPSS service could not provide an ‘appropriate remedy’, since it had no power to address the root causes of Syrian’s suffering. In contrast to MHPSS services, the physical NCD service was tangible, involving medication, medical tests and numerical results. Some patients expressed a preference for this approach, which may have offered them a sense of control in the context of a prevailing sense of hopelessness and limited control over daily life.

Hassan suggests that awareness of mental health and seeking help from MHPSS services is increasing, particularly amongst urban Syrians, and she places more emphasis on stigma as a barrier to help seeking [13]. MSF staff reports of stigma and observation of patients’ desire to be seen as ‘normal’ and not ‘crazy’, are in keeping with other studies of Syrian refugees [11, 13].. Hassan’s describes how emotional suffering is perceived as an inherent aspect of life in Syria, but it is the labelling of distress as ‘psychological’ or ‘psychiatric’ that is a source of shame and fear for the individual and the family [13] . A high perceived level of societal stigma and cultural beliefs about mental health were also found to negatively impact help seeking among Jordanians with mental health problems, outside of the humanitarian context. Most preferred to utilise informal resources rather than see a health professional [47, 46]. Among Jordanian communities, help-seeking is a collective rather than individual enterprise. The stigma of attending MHPSS services would therefore be felt by the whole family, which may further discourage help-seeking [48].

Jordanian doctors’ own beliefs and their gate-keeping role over referral to the MHPSS service may also have contributed to low uptake. They were rooted in both the sociocultural and local medical culture, which may have explained their desire to protect patients from stigmatisation and, their initial distrust in the quality and effectiveness of MHPSS counselling services and their belief that mental illness was the domain of doctors.. The latter has been observed in other settings, where medical professionals have resisted efforts to task shift mental health care to other cadres of staff [49]. The Jordanian doctors’ apparent biomedical approach may also have played a part. The MHPSS approach is relatively new in Jordan, where mental health care has traditionally been delivered by psychiatrists in hospitals [11]. No Syrian health professionals were employed by the clinic due to regulatory restrictions in Jordan. Syrian refugees in Lebanon were found to feel more comfortable receiving care from health professional staff from their own culture who have also endured displacement rather than from local staff [50]. Its is unclear how employing Syrian health professionals would impact on uptake of MHPSS services in this context.

The lack of engagement with MSF MHPSS services may also reflect both Syrians and Jordanian patients’ preferring to use their own culturally-relevant coping mechanisms and resilience and/or that the offered MHPSS services were not culturally-relevant [51–54]. Perception of mental health problems may have been influenced by the Islamic teaching that people should surrender and entrust themselves to the will of an omnipotent God. Hassan notes a view widely held by Syrian Muslims that hardship provides an opportunity to grow and to strengthen one’s faith, which may help them to accept and show patience in the face of harsh reality. However, the notion of human weakness is related also to the idea of *taklif* or entrusting, which she suggests can help to find motivation and drive to cope with hardship [13].

Limited references were made to differences in help seeking by gender in the patient data. This may be because it was not directly enquired about. Staff referred to cultural barriers to women who had experienced GBV accessing MHPSS services, since they were required to attend the clinic accompanied by a male relative. Other work suggests a lower uptake of mental health services by women in Jordan compared to men, despite a higher burden of depression and anxiety [48].

### Implications for integrated MHPSS and NCD services in humanitarian settings

While the link between physical and psychological ill health has been well established in conflict-affected settings, we don’t yet know how best to respond to this duality in a culturally relevant and integrated way. The humanitarian sector has sought to ensure that MHPSS interventions are both evidence-based and culturally relevant by adequately recognising local forms of stress, support and healing [12, 55]. The potential benefits of integrating MHPSS and physical health services have been recognised but there is little practical evidence to guide such integration [29, 56]. Further exploration of how distress is expressed in different populations in relation to personal, cultural and social meanings is required and of how such idioms of distress intersect with physical NCD and psychological symptoms [57, 58].

Lessons learned from the MSF experience in Jordan may be useful in adapting their Irbid programme and in guiding the humanitarian sector more broadly. MSF has implemented evidence-based MHPSS approaches in this context, yet we identified the need for greater engagement with NCD patients to explore how these services could better meet their needs It may be helpful to extend the group “living well sessions”, framed around physical illness, which seemed a more acceptable vehicle to foster problem solving, communication skills and peer support. MSF may also consider expanding psychosocial support services into non-clinical settings, such as community halls, women’s programmes or schools to increase their acceptability and accessibility.

The daily stressors, financial precarity, the myriad barriers to accessing care experienced by Syrian refugee NCD patients in Jordan and the potentially harmful decisions they make as a result of stretched finances have been well documented in our own and others’ studies [45, 59–62]. The free care provided by MSF clearly alleviated much of the NCD-related financial burden affecting their current cohort. However, while we acknowledge the high cost of delivering NCD care, MSF may consider approaches to increase coverage by decentralising aspects of care to the community level, or, indeed, by attempting to address, where possible, some of the socio-politically induced daily stressors, experienced by Syrian refugees and vulnerable Jordanians in Jordan.. Further prospective research is required to design and evaluate culturally relevant approaches to integrating physical and psychological NCD care in humanitarian settings, We advocate taking a holistic approach, addressing NCDs from both health and social policy perspectives and engaging patients in the process from the outset.

### Limitations

Patient participants’ lack of awareness of the MHPSS service prior to interview meant that no conclusions about the patient experience of the actual service could be drawn. As a result, the findings are predominantly characterised by patient preconceptions of what the service might entail and descriptions of their help seeking behaviour. An alternative sampling strategy could have been to purposively sample from users of the MHPSS service, but could have risked masking the wider lack of awareness about the MHPSS service. As the focus of the interviews was on broader programme implementation, using the RE-AIM framework, and not solely on mental health, we did not focus the entire interview on participants’ mental health concerns. However, the framework provided enough flexibility to capture themes related to mental health and the broader socio-political humanitarian environment and served to highlight the importance participants placed on mental health in relation to their NCD management. A greater number of interviews could potentially have been performed but it was felt that theoretical saturation was achieved in relation to our key themes.

## Conclusions

The findings of this study suggest that humanitarian actors must better anticipate mental health needs when designing programmes for patients with NCDs. Furthermore, healthcare providers must examine local perspectives and needs in relation to psychosocial issues and co-design effective, person-centred approaches that are culturally relevant, drawing on pre-existing coping mechanisms and that are acceptable and accessible to both providers and patients.

## Supplementary information

**Additional file 1.**

**Additional file 2.**

**Additional file 3.**

**Additional file 4.**

## Data Availability

The datasets generated and/or analysed during the current study are not publicly available in order to protect participant confidentiality but are available from the corresponding author, with the consent of Médecins sans Frontières, and on reasonable request.

## Abbreviations

CBT: Cognitive Behavioural Therapy
DM: Diabetes Mellitus
HTN: Hypertension
MSF: Médecins sans Frontières
MHPSS: Mental Health & Psychosocial Support Network
NCDs: Non-Communicable Diseases
NGOs: Non-Governmental Organisations
RE-AIM: Reach, Effectiveness, Adoption, Implementation and Maintenance
UNHCR: United Nations High Commissioner for Refugees
WHO: World Health Organization
