# Supplementary material for: “To die is better for me”, social suffering among Syrian refugees at a noncommunicable disease clinic in Jordan: a qualitative study
Source: Confl Health. 2020 Sep 1;14:63. doi: 10.1186/s13031-020-00309-6 (PMC7465779; doi:10.1186/s13031-020-00309-6)
Supplement: Supplementary file 1 — Additional file 1. [file 13031_2020_309_MOESM1_ESM.docx]

**Supplementary Material (SM1): Topic guides**

**Topic guide: semi-structured interviews with NCD patients**

| Key area | Themes | Question |
| --- | --- | --- |
| Introduction | Study aim and agencies involved  Why invited to participate  Consent & any questions? |  |
| Participant Background | Getting to know each other + building rapport | Could you tell us a bit about yourself? *Prompt: e.g. profession, what area live in, when you were first diagnosed with NCD condition?* |
| Reach | Knowledge in community  Access to testing for [NCD condition]  Barriers to testing | What do you know about your condition? *Prompt – e.g. causes, types, who gets it, treatment*  What has been your experience in accessing healthcare and medications for your condition? *Prompt – in Syria, in Jordan, other NGOs or clinics, why choose to come to MSF clinic, does experience differ?*  Do you think a lot of people have your NCD condition in your community?  What do you think prevents people from accessing healthcare/medications for NCD conditions?  What do you think would make it easier for people to access healthcare / medications for NCD conditions?  If MSF were not providing this service what would you do to manage your condition? |
| Adoption and implementation | Information and other support provided  Adjusting to condition | What type of information provided to you about your condition and its treatment when you were enrolled in the MSF clinic? *Prompt: causes, who gets it, chronic nature, medicine types and usage, managing medicines, diet changes, risks and symptoms, frequency of check-ups etc.*  What sources of support did you receive? *Prompt: emotional support from family/friends, information support from health workers, psychosocial support from health workers*  What other support would you have liked to receive?  Do you find it easy to come in to the clinic from the beginning? *Prompt: facilitators or barriers e.g. logistically, socially, culturally*  How has having your NCD condition changed your daily life/routine? *Prompt: e.g. difficulties in changing your daily routine, in Syria or in Jordan?*  What is your experience of the MHPSS part of the programme (group sessions or individual counselling)? |
| Maintenance | Barriers/challenges to adhering to appointments and prescribed medicine/ lifestyle change. | Do you come in regularly for all your appointments? Do you find it easy or difficult to do so? Why? (e.g. travel, time, stigma)  What is your experience when you come to the clinic?  Do you experience any difficulties when you are visiting the clinic for follow up?  What could be done to make it easier for you to come to the clinic?  Do you take your medicines as often as you are prescribed? Why? *(Prompt: don't think it's important, unsure how to take them, can’t read the instructions, too many pills, share with family/ friends, supply rupture)* Do you find it easy or difficult to do so? Why? (*Prompt* *difficult to remember)*  Do you feel any pressure not to take your medicines (*Prompt: stigma from family or community, cost, medication sharing)*  What could be done to make it easier for you to take your medications?  Do you find it easy to maintain the recommended diet, exercise levels, smoking cessation for your condition?  What has helped you to make lifestyle changes? Prompt: health education, medical staff, family or community support?  What challenges do you face in adapting your diet, exercise levels and smoking habits?  Do you thing MHPSS is important?  What challenges do you face in taking part in or attending MHPSS support (group sessions, or individual counselling)? |
| Effectiveness | How coming to the clinic has affected patient's condition | What have been the negative consequences of taking treatment for the condition/ attending the service? *Prompt: physical, psych, costs, time.*  What have been the benefits of receiving treatment for your condition / attending the service? *Prompt: e.g. physical, psychological, social, economic.*  *What have been the positive and negative consequences for you in attending the MHPSS sessions (group or individual counselling)? (Prompt: feel supported, feel better, assists with managing NCD condition, upsetting, difficult)* |
| Thanks and  Close | Anything else to add  Questions/Thanks, feedback info |  |

**Topic guide for focus groups with NCD patients**

| Key area | Themes | Question |
| --- | --- | --- |
| Introduction | Study aim and agencies involved | Why invited to participate? Consent & any questions? |
| Participant Background | Getting to know each other + building rapport | Could you tell us a bit about yourself? *Prompt: e.g. profession, what area live in, when you were first diagnosed with [NCD condition]?* |
| Reach | Access  Barriers to accessing care for NCDs  Ways of reducing barriers | What do you know about your NCD condition(s)? *Prompt – e.g. causes, types, who gets it, treatment*  Could you tell me about how you came to learn about your NCD condition(s)? ? *Prompt – e.g. from friends/family, from the radio (or other media), when diagnosed at hospital.*  What were you told about NCD condition(s) when you were enrolled in the MSF clinic? *Prompt – probe understanding of NCD condition(s) such as causes, risks and its management (medication and diet).*  What do you think might prevent people from attending this service for their NCD condition(s)? *Prompt: lack of knowledge, lack of services, costs, time, quality of services, stigma etc.*  How could access to healthcare for NCD condition(s) be improved? |
| Adoption and implementation | Information  Support | How did you feel when you were enrolled into this clinic? *Prompt: counselling/support experience. Prompt: subsequent days/weeks experience*  Who did you talk to about your experience at the clinic? *Prompt: E.g. family members, friends.*  What were you told about managing your NCD condition(s) after you were enrolled in clinic (by the NCD staff)? *Prompt: medicine types and usage, managing medicines, diet changes, risks and symptoms, frequency of check-ups etc.*  What sources of support did you receive in managing your NCD condition? *Prompt: emotional support from family/friends, information support from health workers, MHPSS from health workers.*  What made it easier for you to access care – initially and continuing care?  What made it difficult for you to access care – initially and continuing care?  What made it easier for you to self-manage your NCD condition at home?  What made it difficult for you to self-manage your NCD condition at home?  How acceptable do you find the NCD service / treatment. *Prompt: e.g. logistically, socially, culturally etc., differences with previous experience of treatment/ service?*  What has been your experience of the psychosocial services offered by the programme – group sessions/individual counselling*?* |
| Maintenance | Challenges  Supportive factors  To support adoption and implementation | What have been the main challenges in maintaining your medical treatment for your NCD condition? *Prompt: time, costs, information, drug supply, pill burden, stigma/shame etc.*  What have been the main challenges in altering your diet? *Prompt: information, costs, support*  What have been the main challenges in increasing your levels of exercise? *Prompt: information, suitable facilities or locations, physical condition, support, costs*  What have been the main challenges in reducing or quitting smoking? *Prompt: information, support, costs, desire*  What could have made accessing care easier for you? *Prompt: e.g. information given – content and way it was delivered; costs; type and quality of care and support; focus on role of the NCD programme/services.*  What could have made achieving lifestyle changes easier for you? *Prompt: e.g. information given – content and way it was delivered; costs; type and quality of care and support; focus on role of the NCD programme/services;*  What support is available to help you to continue to attend the clinic and self-manage your condition?  What additional supports regarding your NCD condition would you like to have? |
| Effectiveness | Unintended consequences  Benefits | What have been the negative consequences of taking NCD treatment / attending the service? *Prompt: physical, psych, costs, time.*  What have been the benefits of receiving NCD treatment / attending the service? *Prompt: e.g. physical, psychological, social, economic.*  What have been the benefits or negative consequences of attending group sessions/ individual counselling? |
| Thanks and  Close | Anything else to add  Questions/Thanks, feedback info | Anything else to add on topic that we haven’t discussed today? Any questions for me? Feedback again on how the discussion will be used and fed back. |

**Topic guide for semi-structured interviews with MSF staff**

| Key area | Themes | Question |
| --- | --- | --- |
| Introduction | Study aim and agencies involved  Why invited to participate  Consent & any questions? |  |
| Participant Background | Getting to know each other + building rapport | Could you tell us a bit about yourself? Prompt: e.g. professional, involvement in the NCD service at Irbid (and previously if relevant)? |
| Reach | Access  Barriers to NCD care provision  Ways of reducing barriers | What are the key challenges for patients to access healthcare (medications, regular clinical review, investigations, interventions) for their NCD condition(s) e.g. knowledge, costs, time, availability or quality of care [expand], stigma etc.  How could access to healthcare for NCD condition be improved? Prompt: improve knowledge (e.g. outreach, radio, health workers etc.), improve availability of services, quality of services etc. |
| Adoption and implementation | Information and support | What types of information are provided to patients when they are enrolled in/ attend the NCD service?  What sources of support are offered to patients when they are enrolled in/ attend the NCD service)?  How acceptable do you think the MSF NCD programme/service, including treatment, is for patients? Prompt: e.g. quality, responsiveness, socially, culturally etc.  What is your experience with implementing the new MSF NCD guideline?  What sources of support and information were available to you to facilitate implementing the guideline? |
| Maintenance | Challenges  Supportive factors  To support adoption and implementation | What do you think are the main challenges facing NCD patients here in terms of managing their condition? Prompt: medicines/testing/attendance - time, costs, information, drug supply etc.; lifestyle changes – knowledge, social/cultural pressures etc.  What could be done to make it easier for NCD patients to access care? Prompt: e.g. information given – content and way it was delivered; costs; type and quality of care and support; [note: focus on role of the [NCD condition] programme/services].  What do you think are the main challenges facing staff here in terms of delivering the NCD care programme in Irbid? Prompt: time, training, clinical support/supervision, guidelines or tools  What could be done to make it easier for staff to deliver this NCD care programme? Prompt: knowledge, time, training, clinical support/supervision, guidelines or tools  What are the benefits of using the MSF NCD guideline?  What are the challenges around using the MSF NCD guideline?  What could be done to facilitate implementation of the guideline? |
| Effectiveness | Unintended consequences  Benefits | What are the benefits of the NCD care programme in Irbid? Prompt: more efficient, less complications, for patients, for staff, for system, for community etc.  What are negative consequences of the NCD care programme in Irbid? Prompt: time, complexity, costs etc. for patients, for staff, for system, for community  What particular aspects of the programme have helped or hindered NCD care? Prompt: clinical aspects, task shifting, introduction of HLO, MHPSS, HV, structures, tools, systems  What particular aspects of the guideline have helped or hindered NCD care? Prompt: supports decision making, ease of use, contradictory, not acceptable to patients, different to usual practice in Jordan. |
