# Supplementary material for: “To die is better for me”, social suffering among Syrian refugees at a noncommunicable disease clinic in Jordan: a qualitative study
Source: Confl Health. 2020 Sep 1;14:63. doi: 10.1186/s13031-020-00309-6 (PMC7465779; doi:10.1186/s13031-020-00309-6)
Supplement: Supplementary file 2 — Additional file 2. [file 13031_2020_309_MOESM2_ESM.docx]

**Supplementary Material 2 (SM2): Semi structured interview participant characteristics**

| **PATIENTS** | | | |
| --- | --- | --- | --- |
| **Code** | **Diagnosis** | **Origin** | **Gender** |
| PT01 | DM, HTN | Jordanian | Male |
| PT02 | DM, HTN | Jordanian | Female |
| PT03 | DM | Syrian | Female |
| PT04 | CVD asthma | Jordanian | Male |
| PT05 | DM, CVD, MH | Syrian | Male |
| PT06 | DM, HTN, | Syrian | Male |
| PT07 | HTN | Syrian | Male |
| PT08 | DM, HTN | Syrian | Male |
| PT09 | DM | Jordanian | Female |
| PT10 | MD, MH, HLO | Syrian | Female |
| PT11 | DM, asthma, MH | Syrian | Female |
| PT12 | DM, CVD | Syrian | Male |
| PT13 | DM (wife of patient) | Jordanian | Female |
| PT14 | DM, HTN | Jordanian | Male |
| PT15 | DM, HTN, CVD, HLO | Syrian | Female |
| PT16 | HTN, CVD, HV | Syrian | Male |
| **STAFF** | | | |
| **Code** | **Position** | | **Gender** |
| ST01 | Clinic staff | | Female |
| ST02 | Clinic staff | | Female |
| ST02 | Administrative staff | | Female |
| ST04 | Clinic staff | | Female |
| ST05 | Clinic staff | | Male |
| ST06 | Clinic staff | | Female |
| ST07 | Clinic staff | | Male |
| ST08 | Clinic staff | | Female |
| ST09 | Clinic staff | | Female |
| ST11 | Management staff (International) | | Female |
| ST12 | Management staff (International) | | Female |
| ST13 | Supervisor (International) | | Female |
| ST14 | Supervisor (International) | | Male |
| ST15 | Supervisor (International) | | Male |
| ST16 | Management staff (International) | | Male |
| ST17 | Management staff (International) | | Male |
| ST18 | Management staff (International) | | Male |
| *Note: Interviews in grey not included as did not contain mental health content*  *CVD=cardiovascular disease; DM=Diabetes Mellitus; HTN=hypertension; MH=mental health; HLO=humanitarian liaison officer; HV=home visit team; LOS= length of stay in Jordan* | | | |
