# Supplementary material for: “To die is better for me”, social suffering among Syrian refugees at a noncommunicable disease clinic in Jordan: a qualitative study
Source: Confl Health. 2020 Sep 1;14:63. doi: 10.1186/s13031-020-00309-6 (PMC7465779; doi:10.1186/s13031-020-00309-6)
Supplement: Supplementary file 3 — Additional file 3. [file 13031_2020_309_MOESM3_ESM.docx]

**Supplementary Material 3 (SM3) : Coding Tree for Thematic Analysis**

| **Theme** | **Category** | **Codes** |
| --- | --- | --- |
| Displacement | Circumstances in Jordan | Safety in Jordan |
|  |  | Settling in Jordan and impact on health |
| Mental Health | Seeking help from mental health services | Unaware of the service |
|  |  | ‘Normal to be anxious’ |
|  |  | Mental health as a private matter |
|  |  | Expected solutions to their distress |
|  |  | Stigma |
|  | Mental health of the Community | High burden of psychological distress in the community |
|  |  | Sources of distress |
|  |  | Coping and support |
|  |  | Hopelessness and fear for the future |
|  |  | (Suicidality and gender-based violence ) |
| Physical Health | Interaction between mental health and physical health | Interconnectedness in explanatory model |
|  |  | Being unable to engage in NCD care due to psychological distress |
|  |  | Burden of psychological distress on patients and impact on NCD care |
