# Supplementary material for: “To die is better for me”, social suffering among Syrian refugees at a noncommunicable disease clinic in Jordan: a qualitative study
Source: Confl Health. 2020 Sep 1;14:63. doi: 10.1186/s13031-020-00309-6 (PMC7465779; doi:10.1186/s13031-020-00309-6)
Supplement: Supplementary file 4 — Additional file 4. [file 13031_2020_309_MOESM4_ESM.docx]

**Supplementary Material 4 (SM4)**

**Language used to describe mental health or related concepts:**

There was some consistency in the language used to describe distress. Anger, tiredness and pressure were all similar language to that recorded by Hassan [59]. However, the authors were unable to assess the consistency of the translation from the original Arabic transcripts. At times, participants could also be seen to be responding to questions by using the same language as the interviewer.

| Anger | ‘*I can be angry maybe 20 times if I face something, I will get angry so these things can’t be accepted’*  *‘We are humans and we can’t stop getting mad’* |
| --- | --- |
| Comfortable or unable to be comfortable | *‘we are psychologically comfortable here’*  *‘we do not feel comfortable in our situation, and our country situation’*  *‘but for us here in Jordan we feel comfortable’*  *‘But I can’t be comfortable but I have to be part of it’*  *‘nobody is comfortable’* |
| Tired | ‘*I’m tired and mentally exhausted… we are all tired as Syrians’*  *‘I really felt tired the most when I came to Jordan because I had no work. I didn’t feel tired but when I came to Jordan and didn’t do anything, I started to feel tired’* |
| Life is not worth living or wanting to die | ‘*I don’t like this life. I would like to die and get rid of it… Every time I come here, they advise me to take care of yourself and I always tell them to die is better for me’* |
| Pressure | ‘there is so much pressure.. I mean the simplest thing is what we faced, it was not easy.’ |
| Anxiety and somatisation | ‘my stomach pain caused by anxiety’  ‘when I am upset I have trembles in my body’ |
